# TGF-β1/Smad Signaling Pathway Regulates Epithelial-to-Mesenchymal Transition in Esophageal Squamous Cell Carcinoma: In Vitro and Clinical Analyses of Cell Lines and Nomadic Kazakh Patients from Northwest Xinjiang, China

**DOI:** 10.1371/journal.pone.0112300

**Published:** 2014-12-02

**Authors:** Lijuan Pang, Qiuxiang Li, Cuilei Wei, Hong Zou, Shugang Li, Weiwei Cao, Jianwei He, Yang Zhou, Xinxin Ju, Jiaojiao Lan, Yutao Wei, Chengyan Wang, Wei Zhao, Jianming Hu, Wei Jia, Yan Qi, Fudong Liu, Jinfang Jiang, Li Li, Jin Zhao, Weihua Liang, Jianxin Xie, Feng Li

**Affiliations:** 1 Department of Pathology and Key Laboratory of Xinjiang Endemic and Ethnic Diseases (Ministry of Education), Shihezi University School of Medicine, Shihezi, Xinjiang, China; 2 Department of Public Health, Shihezi University School of Medicine, Shihezi, Xinjiang, China; 3 Department of Clinical Diagnosis Laboratory, First Affiliated Hospital to Shihezi University School of Medicine, Shihezi, Xinjiang, China; 4 Department of Thoracic and Cardiovascular Surgery, Hospital of Xingjian Production and Construction Corps, Wulumuqi, Xinjiang, China; 5 Department of Pathology, First Affiliated Hospital to Shihezi University School of Medicine, Shihezi, Xinjiang, China; 6 Department of Oncology, People's Hospital of Lianyuan, Lianyuan, Hunan Province, China; Leiden University Medical Center, Netherlands

## Abstract

Invasion and metastasis are the major causes of death in patients with esophageal squamous cell carcinoma (ESCC). Epithelial-mesenchymal transition (EMT) is a critical step in tumor progression and transforming growth factor-β1 (TGF-β1) signaling has been shown to play an important role in EMT. In this study, we investigated how TGF-β1 signaling pathways contributed to EMT in three ESCC cell lines as well as 100 patients of nomadic ethnic Kazakhs residing in northwest Xinjiang Province of China. In vitro analyses included Western blotting to detect the expression of TGF-β1/Smad and EMT-associated proteins in Eca109, EC9706 and KYSE150 cell lines following stimulation with recombinant TGF-β1 and SB431542, a potent inhibitor of ALK5 that also inhibits TGF-β type II receptor. TGF-β-activated Smad2/3 signaling in EMT was significantly upregulated as indicated by mesenchymal markers of N-cadherin and Vimentin, and in the meantime, epithelial marker, E-cadherin, was markedly downregulated. In contrast, SB431542 addition downregulated the expression of N-cadherin and Vimentin, but upregulated the expression of E-cadherin. Moreover, the TGF-β1-induced EMT promoted invasion capability of Eca109 cells. Tumor cells undergoing EMT acquire fibroblastoid-like phenotype. Expressed levels of TGF-β1/Smad signaling molecules and EMT-associated proteins were examined using immunohistochemical analyses in 100 ESCC tissues of Kazakh patients and 58 matched noncancerous adjacent tissues. The results showed that ESCC tissues exhibited upregulated expression of TGF-β1/Smad. We also analyzed the relationship between the above proteins and the patients' clinicopathological characteristics. The TGF-β1/Smad signaling pathway in human Eca109 ESCC cells may carry similar features as in Kazakh ESCC patients, suggesting that TGF-β1/Smad signaling pathway may be involved in the regulation of EMT in ethnic Kazakh patients with ESCC from Xinjiang, China.

## Introduction

Esophageal cancer is the sixth most common cause of cancer-related death worldwide [1]. The incidence and mortality rate of esophageal squamous cell carcinoma (ESCC) is high in nomadic Kazakh minority residing in northwest Xinjiang Province of China [2]. Deep invasion and metastasis remain the leading causes of death for ESCC patients. Therefore, preventing invasion/metastasis is crucial to improve quality of life and survival for patients with ESCC.

Epithelial-to-mesenchymal transition (EMT) plays an important role in cellular transdifferentiation during embryonic development, tumor invasion, and metastasis [3] and is one of the major molecular mechanisms through which invasion and metastasis are promoted during the oncogenic process. EMT is characterized by a breakdown of cell junctions and the loss of epithelial characteristics and cell polarity, leading to cancer progression. Besides the gain of mesenchymal markers, EMT also provides cancer cells with the ability to migrate and invade into surrounding tissues, thereby promoting the subsequent formation of metastases [4]. Although the role of TGF-β1 in induced EMT in cancer progression has been intensively investigated, substantial evidence for the involvement of downstream signaling pathways of TGF-β1 in EMT, especially in the progression of esophageal squamous cell carcinoma, is lacking. TGF-β1 initiates signals by binding to TGF-βRII. Smads are important intracellular effectors of TGF-β1 signaling superfamily [5]. Smad2 and Smad3 mediate signaling by cooperating with Smad4. In contrast, the inhibitory Smad6 and Smad7 inhibit activation of the receptor-regulated Smads.

In this study, we investigated the relationship between TGF-β1/Smad signaling and EMT in ESCC using recombinant TGF-β1 and SB431542, a potent inhibitor of ALK5 that inhibits TGF-β type II receptor, in human ESCC cell lines. We then analyzed the importance of TGF-β1/Smad proteins and EMT proteins in clinical specimens from Kazakh ESCC patients we collected from northwest regions in Xinjiang, China. We present results here showing that TGFβ1/Smad signaling pathway regulates EMT in ESCC cells, in keeping with clinical observations in ethnic Kazakh patients with ESCC.

## Materials and Methods

### Cells lines

Human esophageal carcinoma cell line Eca109 (derived from a Chinese patient with well-differentiated ESCC), KYSE150 (derived from a Japanese patient with poorly differentiated ESCC), and Eca9706 (derived from a Chinese patient with poorly differentiated ESCC) were purchased from the Shanghai Institute of Biochemistry and Cell Biology (Shanghai, China). Cells with 5 passages were maintained in Dulbecco's modified Eagle's medium (DMEM, HyClone Systems, Utah, USA) supplemented with 10% fetal bovine serum (FBS, GIBCO, California, USA), 100 units/mL penicillin and 100 mg/mL streptomycin. Cells were routinely incubated at 37°C under a 5% CO_2_ atmosphere.

### Induction and inhibition of EMT

To induce EMT, cells were seeded into 6-well plates and grown to 70%–80% confluence in complete growth medium. Recombinant human TGF-β1 (R&D Systems, Minnesota, USA) was reconstituted in 4 mM HCl containing 0.1% bovine serum albumin. Cells were then incubated in serum-free medium supplemented with TGF-β1 over the concentration range 0, 1, 5 and 10 ng/mL [6]–[8] at 37°C, 5% CO_2_ atmosphere according to the literature. Cells were harvested at 36 hrs after treatment. An MTT assay for each of the drug is mandatory to evaluate the drug cytotoxicity. All experiments were performed in triplicate and repeated three times.

To inhibit EMT, SB431542 (Sigma Systems, Florida, USA) was dissolved at a concentration of 10 mM in DMSO. Cells were incubated in serum-containing medium supplemented with 0, 1, 5, or 10 µM of SB431542 [9] at 37°C and 5% CO_2_ atmosphere. Cells were harvested at 24 hrs after treatment. All experiments were performed in triplicate and repeated three times. Several dosage groups of SB431542 were designed to evaluate the inhibitory effect on TGF-β1.

### Western blotting analysis

Cells from untreated group, TGF-β1-treated and SB431542-treated cells were washed 3 times with ice-cold PBS. Lysis buffer was then added, and the cells were lysed on ice for 30 min, followed by centrifugation at 12,000 rpm for 10 min at 4°C to remove cell debris. Protein samples were mixed with loading buffer and heated at 100°C for 10 min, after which the samples were separated by 10% SDS-PAGE gels (Bio-Rad, California, USA). Proteins were then transferred to polyvinyl difluoride membranes (Solarbio Systems, Beijing, China). Membranes were blocked in 5% nonfat milk in TBST buffer for 1–2 h at room temperature. The blots were incubated with primary antibodies overnight at 4°C with rabbit anti-p-Smad2 (1∶1000, Cell Signaling Technology, Massachusetts, USA), anti-Smad7 (1∶200, Santa Cruz Biotechnology, California, USA), anti-E-cadherin (1∶500, Abcam, Cambridge, United Kingdom), mouse anti-vimentin (1∶100, Santa Cruz Biotechnology, California USA), or anti-N-cadherin antibodies (1∶1000, Abcam, Cambridge, United Kingdom)This procedure was followed by incubation with sheep anti-mouse (1∶20000) or anti-rabbit (1∶20000) HRP-labeled secondary antibodies for 2 h at room temperature. The bands were detected with an ECL reagent kit (Thermo Systems, Massachusetts, USA). β-Actin immunoblots served as a loading control. All experiments were repeated three times.

### Transwell invasion/metastasis assay

Cell invasion assays were performed using 24-Transwell chambers with 6.5-mm polycarbonate filter membranes (8-µm pore size; Costar, Conning, Connecticut, USA). Each insert was precoated with 60 µL of 8 mg/mL Matrigel (BD Biosciences, New Jersey, USA) and then incubated at 37°C and 5% CO2 for 2–3 hrs. Untreated cells and cells treated with 10 ng/mL TGF-β1 were harvested and suspended in serum-free DMEM. Cell suspensions containing 1×105 cells (100 µL) were plated into the upper chamber of the transwell, and condition medium (600 µl) containing 20% FBS was added to the lower chamber of the transwell. The system was incubated at 37°C, 5% CO_2_ atmosphere for 24 hrs. After incubation, the liquid in the upper chamber was removed, the upper chamber was carefully taken out of the well, and the membrane was fixed with 4% paraformaldehyde for 20 min, Cells that remained on top of the filter were removed and stained with 0.1% crystal violet for 15 min. Staining was terminated by washing with flowing water. Cells that migrated through the membrane were observed under an inverted microscope (Olympus, BX51, Japan). The total number of cells in 5 random visual fields (at 100×) of each well was counted, and the mean was calculated. All experiments were performed at least 3 times and each in triplicate.

### Immunohistochemical (IHC) analyses

One hundred tumor tissue samples and 58 matched control samples (noncancerous adjacent tissues, NCAT) were collected from esophageal cancer patients who had undergone biopsies without radiotherapy or chemotherapy before esophagectomy from the First Affiliated Hospital, Shihezi University School of Medicine; Xinjiang Autonomous Region People's Hospital; and the Friendship Hospital of Yili, all in Xinjiang, China) from 1980 to 2010. All specimens were collected after informed consent was obtained in accordance with our institutional guidelines. The paraffin-embedded tissues were then prepared as a tissue array for IHC analyses. EnVisions two-step immunohistochemical kit (Zhongshan Golden Bridge, Beijing, China) and DAB enhancer (Dako System, Glostrup, Denmark) were used to detect specific target proteins. Briefly, tissue array sections were deparaffinized and rehydrated with ethanol, then quenched with 3% hydrogen peroxide for 20 min. Slides were soaked in 10.0 mM sodium citrate buffer and boiled in a pressure cooker for 8 min at 800 W for antigen retrieval. Slides were incubated with primary antibodies overnight at 4°C with mouse anti-E-cadherin (1∶100, DAKO System, Glostrup, Denmark), anti-vimentin (1∶800, DAKO System, Glostrup, Denmark), anti-N-cadherin (Abcam, Cambridge, United Kingdom), rabbit anti-TGF-β1 (1∶100, Sc-146-R, Santa Cruz Biotechnology, California, USA), anti-TGF-βRII (1∶100, Sc-220-R, Santa Cruz Biotechnology, California, USA), or anti-p-Smad2/3 (1∶100, Sc-11769-R, Santa Cruz Biotechnology, California, USA), respectively (Table S1 in File S1). The samples were then washed with PBS and subsequently incubated with secondary antibodies for 30 min. The sections were visualized by incubating with diaminobenzidine tetrahydrochloride and counterstained with hematoxylin. PBS was used to replace the primary antibody as negative control.

Assessment of immunostaining was carried out by three experienced pathologists, who were blind to patient IDs, and any differences in interpretation were resolved by consensus. Brownish-yellow staining appearing in the cell membrane, cytoplasm, and/or nucleus was considered as positive staining. The samples were scored according to the staining intensity and the ratio of positive cells as follows: <5% positive cells, 0; 6%–25% positive cells, 1; 26%–50% positive cells, 2; 51%–75% positive cells, 3; and >76% positive cells, 4. In terms of the staining intensity, no staining was recorded as 0 (negative), pale yellow was recorded as 1 (weakly positive), brownish-yellow was recorded as 2 (positive), and brown was recorded as 3 (intensive positive). The product of the two scores determined the final score values; scores of 0–1 were regarded as negative (−), while scores of 2 or more were regarded as positive [10]. For p-Smad2/3 evaluation, we counted the number of p-Smad2/3-positive nuclei relative to the total number of the nuclei in a field.

### Statistical analysis

All data were statistically analyzed using SPSS17.0 software package. Quantitative data were presented as mean±SE. Differences in means, positive rates and positive staining intensity were analyzed by ANOVA, Chi-Square test and Mann-Whitney U test, respectively. McNemar-Bowker Test was used to compare differences of the expression of TGF-β1, P-Smad2/3, TGFβRII, N-cadherin, Vimentin, E-cadherin between the tumor and the noncancerous adjacent tissue of the same patients. Interval by interval Pearson correlation was used to analyze the correlation between the TGF-β1 and P-Smad2/3, TGFβRII, E-cadherin, N-cadherin, Vimentin. P-values of less than 0.05 were considered statistically significant.

### Ethics statement

This study was approved by the Institutional Ethics Review Board at the First Affiliated Hospital, Shihezi University School of Medicine. Written informed consent was obtained from all patients. All specimens were handled and made anonymous according to the ethical and legal standards. No information was provided in regard with the therapeutic regimen and patient outcome and survival, although this infomation must be available as the cohort had been treated from 1980-2010. we included all Kazakh ESCC cases collected from 4 distant hospitals.

## Results

### TGF-β1 induces morphological changes in Eca109 and KYSE150 cells

**Figure 1:**
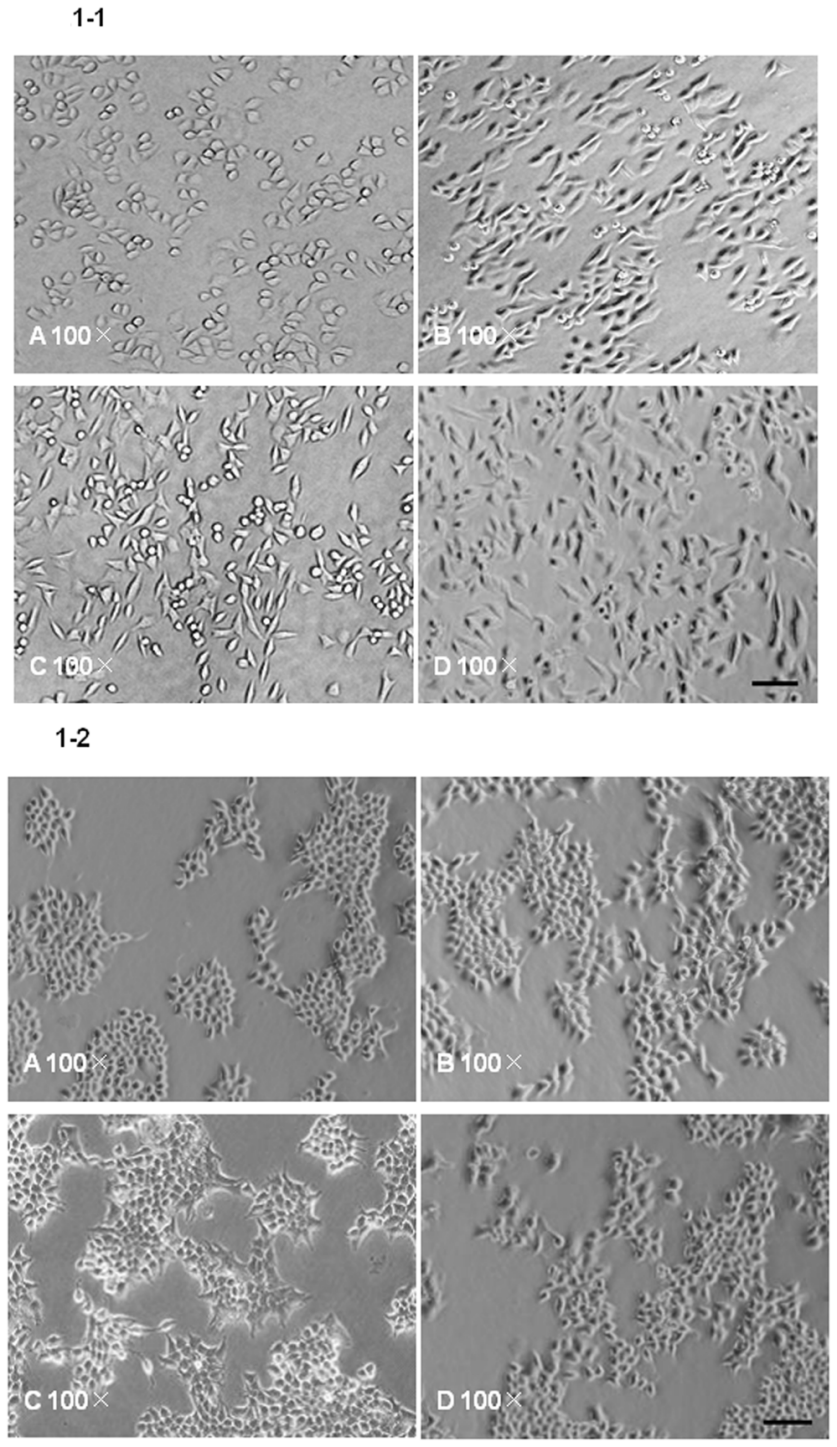
Morphological changes of Eca109(1–1) and KYSE150(1–2) cells after TGF-β1 treatment. A: Untreated Eca109 and KYSE150 cells, 100×; B,C: Eca109 and KYSE150 cells treated with TGF-β1 (1,5 ng/mL) for 36 hrs, 100×. D Control stimulation with TGF-β1 (5 ng/mL) in the presence of SB4315425(5 µM) for 36 hrs, 100×. Scale bars represent 50 µm.

To investigate the effects of TGF-β1 on the activity of Eca109 and KYSE150 cells, we treated cells with 0, 1, 5, 10 ng/mL of TGF-β1, of which 5 ng/mL served as a control dosage, in the presence of SB431542 (5 µM) for 36 hrs. Untreated cells exhibit typical epithelial morphology (Fig. 1–1A, 1–2A). After 36 hrs of 1 ng/ml TGFβ1 treatment, Eca109 and KYSE150 cells lost the epithelial morphology and acquired mesenchymal traits showing fibroblast-like appearance (Figure. 1–1B, 1–2B). Cells treated with 5 ng/ml TGF-β1 showed much more obviously elongated spindle-like shape (Figure. 1–1C, 1–2C). Interestingly, SB431542 treatment attenuated the effect of TGF-β1 without obvious morphologic alterations on ESCC cells (Figure 1–1D, 1–2D).

**Figure 2:**
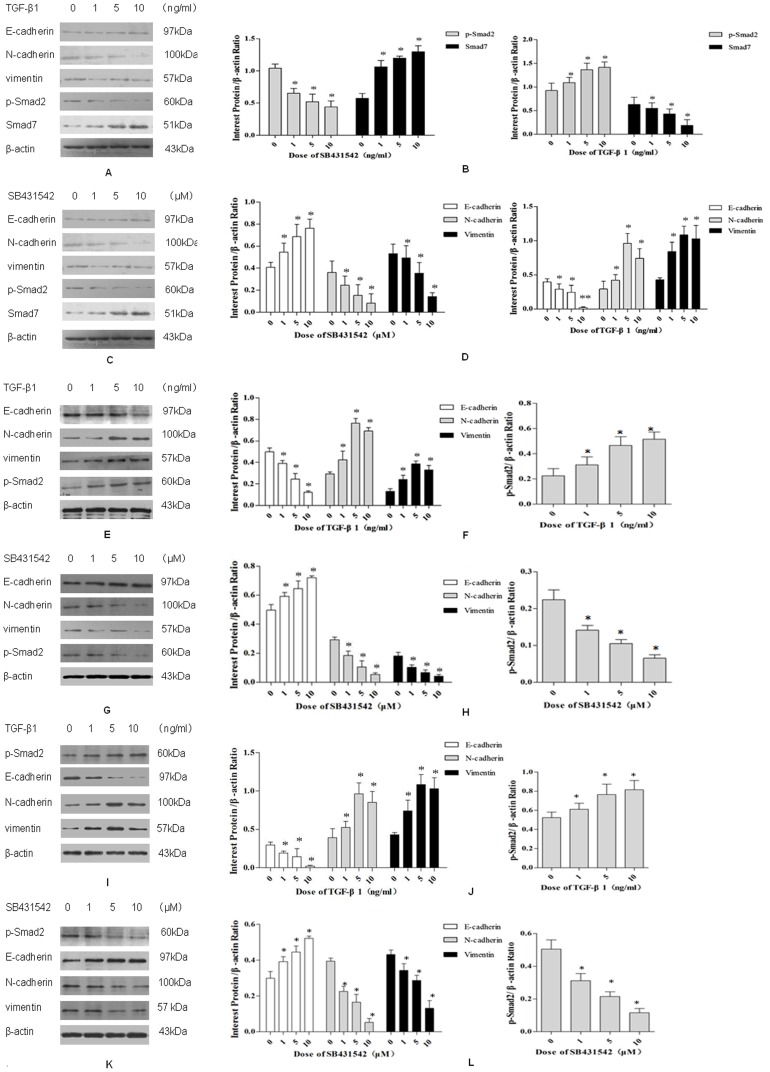
Western blot analysis of E-cadherin, N-cadherin, vimentin, P-Smad2 protein expression in Eca109(2A–D), Eca9706(2E–H), KYSE150(2I–L) cells treated with TGF-β1 or SB431542 at differential concentrations. **A**. Effects of treatment of Eca109 cells with TGF-β1 (1, 5, or 10 ng/mL) on the expression of E-cadherin (molecular weight 97 kDa), N-cadherin (molecular weight 100 kDa), vimentin (molecular weight 57 kDa), p-Smad2 (molecular weight 52 kDa) and Smad7 (molecular weight 51 kDa) by Western Blot. **B**. Quantiative analysis of treatment of Eca109 cells with TGF-β1 (1, 5, or 10 ng/mL), E-cadherin, N-cadherin, vimentin, P-Smad2 and Smad7 expression levels; Y axis: banding densities of test marker versus β-actin. Data are expressed as a significant change relative to the control. Each bar represents the mean±s.d. *, p<0.05, **, p<0.01. **C**. Effects of SB431542 (1, 5, or 10 µM) on the expression of E-cadherin, N-cadherin, vimentin, Smad2/3 and Smad7 in Eca109 cells by Western Blot. **D**. Quantiative analysis of treatment of Eca109 cells with SB431542 (1, 5, or 10 µM), E-cadherin, vimentin, Smad2/3 and Smad7 expression levels; Y axis: banding densties of test versus β-actin. Data are expressed as a significant change relative to the control. Each bar represents the mean±s.d. *, p<0.05, **, p<0.01; Each experiment was repeated three times. **E**. Effects of treatment of Eca 9706 cells with TGF-β1 (1, 5, or 10 ng/mL) on the expression of E-cadherin, N-cadherin, vimentin, p-Smad2 by Western Blot. **F**. Quantiative analysis of treatment of Eca 9706 cells with TGF-β1 (1, 5, or 10 ng/mL), E-cadherin, N-cadherin, vimentin, P-Smad2 expression levels; Y axis: banding densities of test marker versus β-actin. Data are expressed as a significant change relative to the control. Each bar represents the mean±s.d. *, p<0.05, **, p<0.01. **G**. Effects of SB431542 (1, 5, or 10 µM) on the expression of E-cadherin, N-cadherin, vimentin, Smad2/3 in Eca 9706 cells by Western Blot. **H**. Quantiative analysis of treatment of Eca 9706 cells with SB431542 (1, 5, or 10 µM), E-cadherin, vimentin, Smad2/3 expression levels; Y axis: banding densties of test versus β-actin. Data are expressed as a significant change relative to the control. Each bar represents the mean±s.d. *, p<0.05, **, p<0.01; Each experiment was repeated three times. **I**. Effects of treatment of KYSE150 cells with TGF-β1 (1, 5, or 10 ng/mL) on the expression of E-cadherin, N-cadherin, vimentin, p-Smad2 by Western Blot. **J**. Quantiative analysis of treatment of KYSE150 cells with TGF-β1 (1, 5, or 10 ng/mL), E-cadherin, N-cadherin, vimentin, P-Smad2 expression levels; Y axis: banding densities of test marker versus β-actin. Data are expressed as a significant change relative to the control. Each bar represents the mean±s.d. *, p<0.05, **, p<0.01. **K**. Effects of SB431542 (1, 5, or 10 µM) on the expression of E-cadherin, N-cadherin, vimentin, Smad2/3 in KYSE150 cells by Western Blot. **L**. Quantiative analysis of treatment of KYSE150 cells with SB431542 (1, 5, or 10 µM), E-cadherin, vimentin, Smad2/3 expression levels; Y axis: banding densties of test versus β-actin. Data are expressed as a significant change relative to the control. Each bar represents the mean±s.d. *, p<0.05, **, p<0.01. Each experiment was repeated three times.

### TGF-β1/Smad signaling induces EMT in different ESCC cell lines

Different doses were selected according to published papers [6]–[9]. MTT experiment showed no drug cytotoxicity at 0, 1, 5, 10, 15 ng/ml TGF-β1 treatment compared with controls (Fig. S1 in File S1). To determine whether TGF-β1/Smad signaling was involved in the EMT, Western blotting analysis was conducted to detect changes among key molecules involved in EMT. TGF-β1 decreased the expression of E-cadherin, but increased the expression of N-cadherin and Vimentin in Eca109, KYSE150 and Eca9706 cells (Fig. 2A–L), which was consistent with the typical changes during EMT. Furthermore, TGF-β1 also up-regulated p-Smad2 levels, and down-regulated Smad7 levels in the cells (Fig. 2A). Band densitometry analysis confirmed these changes to be significantly altered upon TGF-β1 treatment (Fig. 2B, *P*<0.05, Y axis: optical densities of test bands versus β-actin bands), further suggesting that TGF-β1 signaling pathway is involved in the EMT process in ESCC cells (Fig. 2 E, F, I, and J).

To further confirm the essential role of TGF-β1 and its downstream Smads in EMT, we treated the cells with SB431542. After SB431542 treatment, E-cadherin expression increased, Vimentin and N-cadherin expression decreased (Fig. 2C, G., K). We evaluated the E-cadherin, Vimentin expression in response to SB431542 treatment, E-cadherin and vimentin protein levels were significant differences in the SB431542 treated ESCC cells as compared with controls, (Fig.2D, H, L, *p*<0.05, Y axis: optical densities of test bands versus β-actin bands). Furthermore, Western blot analysis showed that p-Smad2 expression were down-regulated after treatment with SB431542 (Fig. 2C, G., K), Band densitometry analysis showed significant differences in SB431542-treated ESCC cells as compared with controls (Fig. 2 D, H, L, *P*<0.05). Thus, SB431542 may inhibit EMT process by blocking TGFβ1/Smad signaling pathway. We further explored the role of SB431542 in blocking TGFβ1-induced EMT and found that SB431542 could attenuate TGF β1-mediated EMT in a dose-dependent manner (Fig. S2 in File S1).

### TGF-β1-induced EMT promotes tumor invasion

**Figure 3:**
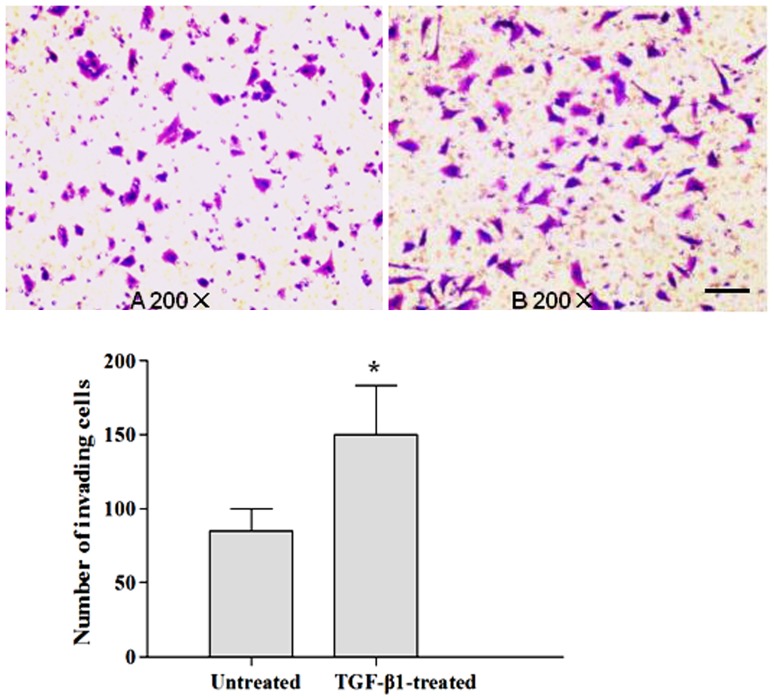
Transwell assay of Eca109 cells with or without TGF-β1 treatment. A: Untreated Eca109 cells, 200×. B: Eca109 cells treated with TGF-β1 (10 ng/mL) for 24 h, 200×. C: The number of invading cells: Untreated Eca109 cells vs. Eca109 cells treated with TGFβ1. Data shown represent the average of 3 independent experiments. *p <0.001, compared with untreated cells. All experiments were performed at least 3 times in triplicate. Scale bars represent 100 µm.

We used transwell invasion assays to investigate the effects of TGF-β1-induced EMT on invasion in ESCC cells. Five random fields were counted per transwell membrane. Treatment with TGF-β1 resulted in a significant increase in the number of cells that invaded through the membrane (141.73±40.81 for TGF-β1-treated cells versus 84.80±20.06 for untreated control cells; *P*<0.001; Fig. 3). Thus, these results suggested that the TGF-β1-induced EMT promoted the invasion capability of Eca109 cells.

### IHC analyses of TGF-β1, TGF-βRII, and p-Smad2/3 proteins and their correlation with clinicopathological features in ESCC patients

Clinicopathological characteristics and demographics of the patients are shown in Table 1. The patients consisted of 64 males and 36 females, aged from 34 to 76 years with a mean age of 58.8±9.0. All the cases were confirmed by histopathological diagnoses as squamous cell carcinoma by two independent pathologists. Carcinomas were classified and staged based on World Health Organization criteria [11]. Among these 100 ESCC cases, 26 were well differentiated, 63 were moderately differentiated, and 11 were poorly differentiated. In these cases, 54 had metastasis in lymph nodes (LN), 57 were found infiltrated to submucosa or to the muscular layer, and 43 were found infiltrated to the deep mantle layer.

**Table 1:** TGF-β1/Smad signaling pathway protein expression in relation to clinical pathological characteristics in Kazakh ESCC.

Clinicopathological features	N	TGF-β1			*P*	TGFβRII			*P*	p-Smad2/3			*P*
		−	+	++/+++		−	+	++/+++		−	+	++/+++	
Normal	58	30	22	6	<0.001	9	38	11	0.056	24	20	14	<0.001
ESCC	100	8	28	64		29	28	43		15	27	58	
Sex													
Male	64	4	18	42	0.454	19	19	26	0.840	12	16	36	0.161
Female	36	4	10	22		10	9	17		3	11	22	
Age (y)													
<60	51	5	13	33	0.746	12	15	24	0.219	9	17	25	0.449
≥60	49	3	15	31		17	13	19		6	10	33	
Tumor invasion													
Superficial layer	57	5	15	37	0.999	15	14	28	0.496	7	16	34	0.381
Deep layer	43	3	13	27		14	14	15		8	11	24	
LN metastases													
Yes	54	4	13	37	0.999	16	14	24	0.880	9	13	35	0.799
No	46	4	15	27		13	14	19		6	14	23	
Tumor differentiation													
Well	26	3	11	12	0.033	5	5	16	0.202	5	11	10	0.034
Moderate-poor	74	5	17	52		24	23	27		10	16	48	
Stage													
I+II	75	8	19	48	0.195	24	21	30	0.252	12	22	41	0.755
III	25	0	9	16		5	7	13		3	5	17	

**Figure 4:**
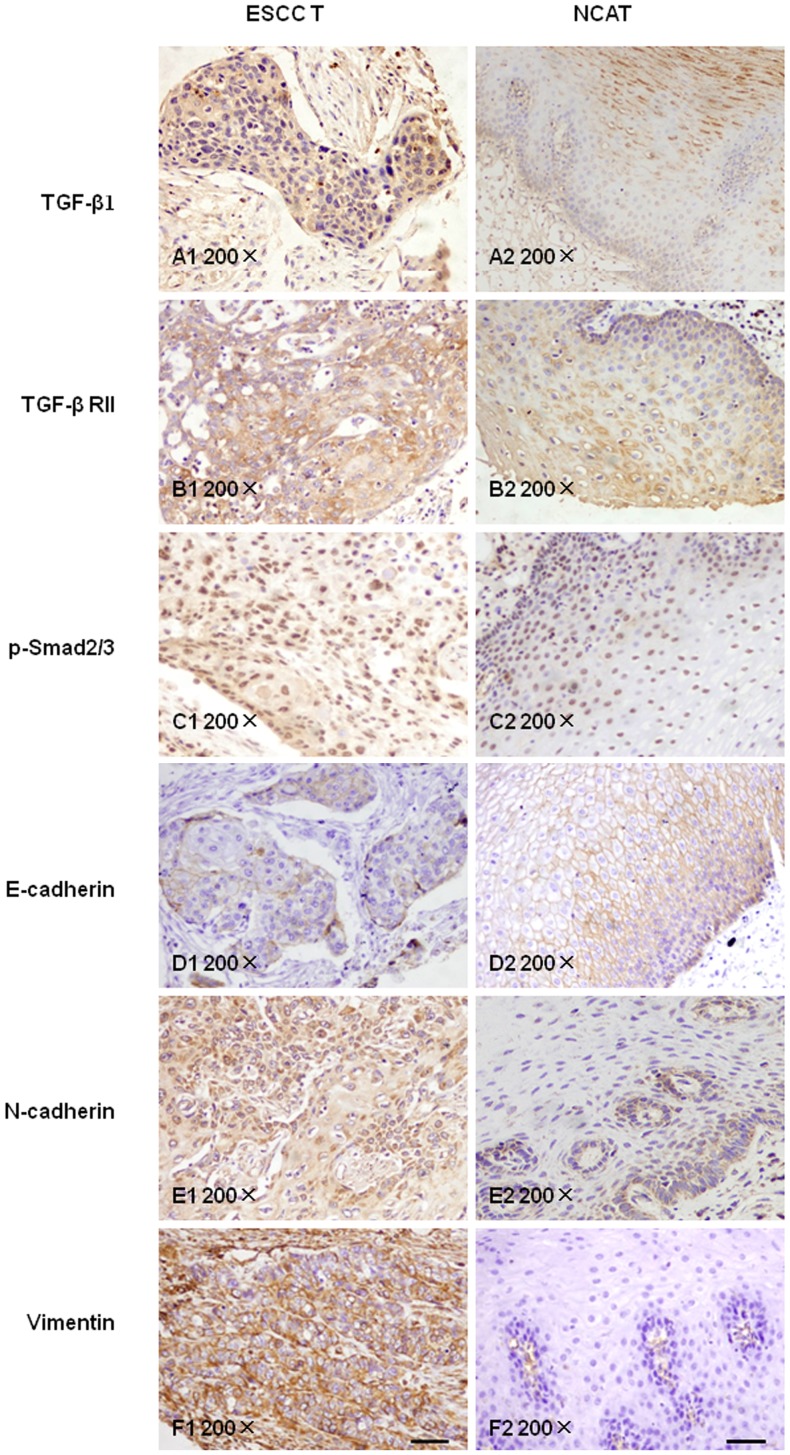
Representative immunohistochemical staining for TGF-β1 (A1,A2), TGF-β RII (B1,B2), p-Smad2/3 (C1,C2), E-cadherin(D1,D2), N-cadherin(E1,E2), and vimentin(F1,F2) in ESCC and NCAT tissues (200×). Scale bars represent 100 µm.

Representative IHC staining images of TGF-β1, TGF-βRII, and p-Smad2/3 are shown in Fig. 4. Positive expression of TGF-β1 was mainly located in the cytoplasm (Fig. 4A). The expression of TGF-βRII was observed in the membrane (Fig. 4B), while p-Smad2/3 expression was observed in the nucleus (Fig. 4C).

The relationships between clinicopathological characteristics and the expression of TGF-β1, TGF-βRII, and p-Smad2/3 are shown in Tables 1 and 2. Positive expression of TGF-β1 was observed in 92% (92/100) of tumor specimens and 48.3% (28/58) of adjacent tissues (*P*<0.001). The expressed levels of TGF-β1 were not correlated with sex, depth of invasion, LN metastasis, pathological stage, tumor differentiation, or distant metastasis (*P*>0.05). However, the ratio of strong positive staining was significantly higher in moderately to poorly differentiated tumors (70.3%) than in well-differentiated tumors (46.2%).

**Table 2:** The comparison between the tumor and the adjacent tissue of the same patients.

NORMAL	ESCC											
	TGF-β1				P-SMAD2/3				TGFβR II			
	−	+	++	P	−	+	++	P	−	+	++	P
−	2	9	19	<0.001	1	7	16	<0.001	6	2	1	0.601
+	1	1	20		5	2	13		2	32	2	
++	2	4	0		1	13	0		1	4	9	

TGF-βRII positive staining did not differ significantly between ESCC tissues (71/100, 71%) and adjacent normal tissues (49/58, 84.5%; *P* = 0.056). Moreover, its expression was not correlated with patient age, differentiation, pathologic stage, or LN metastasis (*P*> 0.05). The ratio of strong positive staining was significantly higher in well-differentiated tumors (61.5%) than in moderately to poorly differentiated tumors (36.5%).

The expression of p-Smad2/3 differed significantly between ESCC samples (85/100, 85%) and adjacent tissues (34/58, 58.6%; *P*<0.001). However, its expression was not correlated with patient age, differentiation, pathological stage, and LN metastasis (*P*>0.05). The ratio of intense positive staining was significantly higher in moderately to poorly differentiated tumors (64.9%) than in well-differentiated tumors (38.5%).

Of 92 cases of ESCC that expressed TGF-β1 protein, 80 expressed p-Smad2/3 protein, and 68 expressed TGF-βRII protein. The expression of TGF-β1 was positively correlated with the expression of p-Smad2/3 (*r* = 0.186, *P* = 0.064, Table 3). Although the expression of TGF-βRII was not correlated with clinicopathological features in tumor tissues (*P*>0.05), the expression of TGF-βRII was positive correlated with the overexpression of TGF-β1 (*r* = 0.218, *P*<0.05, Table 3). These data suggested that the TGF-β1 signaling pathway may regulate ESCC in Kazakh patients from Xinjiang, China.

**Table 3:** Correlation between the expression of TGF-β1 protein and TGF-βRII/p-Smad2/3 proteins in ESCC.

TGF-β1	TGF-βRII			p-Smad2/3		
	−	+	Total	−	+	Total
−	5	3	8	3	5	8
+	24	68	92	12	80	92
Total	29	71	100	15	85	100

### Expressed levels of E-cadherin, N-cadherin, and vimentin and their correlations with clinicopathological features

The expression of E-cadherin was observed in the membrane of cancer cells in 20% (20/100) of tumor tissues (Fig. 4D) and in 53.4% (31/58) of noncancerous tissues; the difference between tumor and control tissues was statistically significant (*P*<0.001, Tables 4 and 5). Moreover, the expression of E-cadherin was correlated with differentiation (*P*<0. 05, Table 4), but was not correlated with sex, depth of invasion, pathological stage, and LN metastasis (*P*>0.05, Table 4).

**Table 4:** EMT protein expression in relation to clinical pathological characteristics in Kazakh ESCC.

Clinicopathological features	N	E-cadherin		*P*	N-cadherin		*P*	Vimentin		*P*
		Negative	Positive		Negative	Positive		Negative	Positive	
Normal	58	27	31	0.001	27	31	0.001	58	0	0.001
ESCC	100	80	20		8	92		70	30	
Sex										
Male	64	49	15	0.252	5	59	0.999	43	21	0.413
Female	36	31	5		3	33		27	9	
Age (y)										
<60	51	37	14	0.057	6	45	0.269	33	18	0.239
≥60	49	43	6		2	47		37	12	
Tumor invasion										
Superficial layer	57	43	14	0.189	5	52	0.999	44	13	0.071
Deep layer	43	37	6		3	40		26	17	
LN metastases										
Yes	54	41	13	0.270	3	51	0.715	38	16	0.930
No	46	39	7		5	41		32	14	
Tumor differentiation										
Well	26	17	9	0.030	5	21	0.027	17	9	0.551
Moderate-poor	74	63	11		3	71		53	21	
Stage										
I+II	75	62	13	0.248	8	67	0.195	50	25	0.208
III	25	18	7		0	25		13	12	

**Table 5:** The comparison between the tumor and the adjacent tissue of the same patients.

NORMAL	ESCC								
	E-cadherin			N-cadherin			Vimentin		
	−	+	P	−	+	P	−	+	P
−	20	7	0.016	19	8	0.008	24	33	<0.001
+	25	6		26	5		1	0	

Yellow-brown particles in the cell membrane/cytoplasm were considered as positive staining. Positive expression of N-cadherin was mainly observed in the cytoplasm of tumor tissues (Fig. 4E). The positive expression of N-cadherin protein was observed in 92% (92/100) of tumor tissues, but only 53.4% (31/58) of noncancerous tissues (*P*<0.001, Table 4). Moreover, N-cadherin expression was correlated with differentiation (*P*<0.05), but not with other clinicopathological characteristics (*P*>0.05, Table 4).

Vimentin was expressed in the cytoplasm of ESCC samples (Fig. 4F). The positive expression of vimentin protein was observed in 30% (30/100) of tumor tissues, but 0% (0/58) of noncancerous tissues (*P*<0.001, Table 4). However, its expression was not correlated with other clinicopathological characteristics (*P*>0.05, Table 4).

Of the 80 cases that were negative for E-cadherin expression, 72 were positive for N-cadherin and 25 were positive for vimentin. A negative correlation was found between E-cadherin and N-cadherin expression (*r* = −0.231, *P*<0.05, Tables 5 and 6). However, there was no significantly negative correlation between E-cadherin and vimentin expression (*r* = −0.055, *P* = 0.590, Table 6). Taken together, these data suggested that EMT may contribute to the progression of ESCC in Kazakh patients.

**Table 6:** Correlation between the expression of E-cadherin protein and N-cadherin/vimentin proteins in ESCC.

E-cadherin	N-cadherin			vimentin		
	−	+	Total	−	+	Total
−	8	72	80	55	25	80
+	6	14	20	15	5	20
Total	14	86	100	70	30	100

*r* = −0.231, *p* = 0.021 vs. N-cadherin; r = −0.055, *p* = 0.590 vs. vimentin.

### Correlations of key molecules in the TGF-β1 signaling pathway with EMT-associated protein in carcinoma tissues

Of the 92 cases that were positive for TGF-β1, 16 were positive for E-cadherin expression, 87 cases were N-cadherin-positive, and 29 cases were N-cadherin-positive. The expression of TGF-β1 was negatively correlated with E-cadherin (*r* = −0.221, *P*<0.05, Tables 5 and 7), and positively correlated with N-cadherin (*r* = 0.321, *P* = 0.001) and vimentin (*r* = 0.113, *P* = 0.265), respectively (Table 7). Thus, these data suggested that the TGF-β1/Smad signaling pathway may be involved in the regulation of EMT in Kazakh ESCC patients.

**Table 7:** Correlation between the expression of TGF-β1 protein and E-cadherin/N-cadherin/vimentin proteins in ESCC.

TGF-β1	E-cadherin			N-cadherin			vimentin		
	−	+	Total	−	+	Total	−	+	Total
−	4	4	8	3	5	8	7	1	8
+	76	16	92	5	87	92	63	29	92
Total	80	20	100	8	92	100	70	30	100

*r* = −0.221, *p* = 0.027 vs. E-cadherin; r = 0.321, *p* = 0.001 vs. N-cadherin; r = 0.113, *p* = 0.265 vs. vimentin.

## Discussion

The incidence of esophageal cancer varies depending on countries, geographic regions, and races [1]. In Kazakh minorities residing in northwest Xinjiang, China, the incidence and mortality of ESCC are high with the latter being 69 per 100,000, much higher than the national average of ESCC mortality in China (15 per 100,000) and other ethnic groups residing in the same region (22.3 per 100,000) [2]. ESCC is often diagnosed at later stages when invasion and metastasis occur. Thus, late diagnosis of ESCC is the main culprit for the ineffective treatment, and deep invasion and metastasis remain the leading causes of death among Kazakh patients with ESCC.

It is well established that EMT is closely related to tumor's invasion and metastasis [12], [13]. EMT-inducing factors are mainly derived from stimuli outside of the primary tumor, such as inflammatory cytokines, cell growth factors and hypoxia among others. Among these factors, the role of growth factors in the induction of EMT in tumor cells is the most concerned and studied [14], [15]. TGF-β1 is regarded as the key growth factor involved in driving EMT [16]. Moreover, TGF-β1 has been reported to induce EMT in multiple cancer cell lines [17]–[20]. TGF-β1 can induce signals through Smad pathway. Activated TGF-β1 exerts its biological effects through the activation of its downstream mediators, Smad2 and Smad3. The activated Smad2/3 forms a heteromeric complex with Smad4 and translocates into the nucleus to induce the transcription of target genes, Smad7 which is an antagonist for TGF-β-activated signaling pathway that negatively regulates the transcription of TGF-β target genes. TGF-β1 can send signals through non-Smad pathways including ERK, MAPK, PI3K, etc. Many studies have explored roles of TGF-β1-activated Smads in the EMT. However, until now, the role of TGF-β/Smad signaling pathway in the regulation of EMT in Kazakh patients with ESCC has not been reported.

In this study, several ESCC cell lines treated with varying doses of TGF-β1 have shown typical EMT characteristics including morphologic changes and expression profiles. Our data have demonstrated that recombinant TGF-β1, either in well or poor differentiated ESCC cells, may induce morphologic changes from cuboidal to spindle shape. Interestingly, this morphologic change did not occur obviously in the presence of SB431542 when stimulated with TGF-β1, suggesting an effective blockage of TGF-β1 by SB-431542. After TGF-β1 treatment, ESCC cells exhibit decreased expression of epithelial markers and increased expression of mesenchymal markers, of which E-cadherin is down-regulated while N-cadherin and Vimentin are up-regulated. These alterations are in agreement with the EMT phenotype reported by Ohashi et al [21]. Furthermore, we have also observed upregulated p-Smad2 expression and downregulated Smad7 expression in response to TGF-β1 treatment.

Our results have shown that TGF-β1 is able to activate Smads in EMT in ESCC cells. We further use SB431542 to treat cells aiming to clarify the requirement of TGFβ-Smad signaling pathway in EMT progression. As expected, the inhibitor is able to efficiently inhibit the endogenous TGF-β1 activation and dramatically attenuate EMT process indicated by the downregulation of vimentin and N-cadherin expression. We have also found that TGF-β1-induced EMT can be blocked by SB432542. SB432542 abrogates the function of TGF-β1-induced EMT in keeping with a previous report [9]. Therefore, TGF-β signaling is required for the induction of EMT in esophageal cancer cells.

It is known that Smad7, a negative regulator of TGF-receptor I (TBRI) kinase, disrupts the activation of Smad2 and Smad3 and restrains TGF-β-mediated Smad signaling [22]–[23]. Pi et al. [24] have showed that epigallocaechin-3-gallate (EGCG) can reverse the TGF-β1-mediated process of EMT by inducing Smad7 upregulation. Our current study demonstrate that TGF-β1 treatment may activate the canonical TGF-β1 signaling by the induction of phosphorylated smad2/smad3, while Smad7 inhibits such activation, which can enhance the EMT-associated invasive phenotypes of ESCC cells in vitro.

IHC studies on ESCC patient tissues have demonstrated increased expression of TGF-β1 and p-Smad2/3 as compared with non-cancerous adjacent tissues (*P*<0.001). On the contrary, positive IHC rate of TGFβRII is higher in non-cancerous adjacent tissues than those in ESCC tissues (*P*>0.05). Although no significant correlations are found between expressed levels and clinicopathologic characteristics, the intensities of positive staining may be different among cell differentiation categories. Zhou et al. [25] have reported that immunostaining of TGF-β1 is increasing with the degree of the cancer lesion, intensifying from normal to basal cell hyperplasia (BCH) to dysplasia (DYS) to carcinoma in situ (CIS) and to squamous cell carcinoma (SCC) (*P*<0.05). Our observations suggest that TGF-β1 signaling pathway may play a role important in Kazakh patients with ESCC.

We have, for the first time, examined the correlations between expressed TGF-β1 signaling molecules and EMT progression in ESCC, aiming to identify potential molecules as specific markers involved in the carcinogenesis of this particular cancer. In Kazakh ESCC, the levels of EMT-associated proteins, such as E-cadherin, N-cadherin, and Vimentin, are significantly correlated with lymph node metastasis and distant metastasis as evidenced by our IHC analyses and others' [26], [27]. It is interesting to note that the expressed levels of TGF-β1 are negatively correlated with the levels of E-cadherin, but positively correlated with those of N-cadherin and vimentin. Our observations demonstrate that TGF-β1 signaling may be a pathway involved in the induction of EMT in Kazakh patients with ESCC.

In conclusion, we have demonstrated that TGF-β1 triggers the Smad-dependent signaling pathway which may be involved in the regulation of EMT in Kazakh ESCC patients. EMT is a critical step in the progression of cancer. Our observations, therefore, warrant further studies to elucidate the mechanism(s) of EMT in ESCC and to identify potential targets through which EMT may be blocked in ESCC.

## Supporting Information

File S1 **Table S1,** Dilution, pretreatment, immunostaining, positive controls, and source for the primary antibodies. **Figure S1,** ESCC cells treated with TGF-β1 (0, 1, 5, 10, 15 ng/mL) for 36 hrs. Quantitative analysis of MTT showed no significant differences among different doses. **Figure S2,** Western blot analysis of E-cadherin, N-cadherin, vimentin, P-Smad2 protein expression in ESCC cells treated with 5ng/ml TGF-β1 in the presence of 0, 1, 5, 10 µM SB431542. **M.** Effects of treatment of Eca109 cells with TGF-β1 (1, 5, or 10 ng/mL) on the expression of E-cadherin (molecular weight, 97 kDa), N-cadherin (molecular weight, 100 kDa), vimentin (molecular weight, 57 kDa), p-Smad2 (molecular weight, 52 kDa) and Smad7 (molecular weight, 51 kDa) by Western Blots. **N.** Quantitative analysis of treatment of Eca109 cells with TGF-β1 (1, 5, or 10 ng/mL), E-cadherin, N-cadherin, vimentin, P-Smad2 and Smad7 expression levels; Y axis: banding densities of test marker versus β-actin. Data are expressed as a significant change relative to the control. Each bar represents the mean±s.d. *, *P*<0.05, **, *P*<0.01.
(DOC)
