# Supplementary material for: TGF-β1/Smad Signaling Pathway Regulates Epithelial-to-Mesenchymal Transition in Esophageal Squamous Cell Carcinoma: In Vitro and Clinical Analyses of Cell Lines and Nomadic Kazakh Patients from Northwest Xinjiang, China
Source: PLoS One. 2014 Dec 2;9(12):e112300. doi: 10.1371/journal.pone.0112300 (PMC4251902; doi:10.1371/journal.pone.0112300)
Supplement: File S1 — Table S1, Dilution, pretreatment, immunostaining, positive controls, and source for the primary antibodies. Figure S1, ESCC cells treated with TGF-β1 (0, 1, 5, 10, 15 ng/mL) for 36 hrs. Quantitative analysis of MTT showed no significant differences among different doses. Figure S2, Western blot analysis of E-cadherin, N-cadherin, vimentin, P-Smad2 protein expression in ESCC cells treated with 5ng/ml TGF-β1 in the presence of 0, 1, 5, 10 µM SB431542. M. Effects of treatment of Eca109 cells with TGF-β1 (1, 5, or 10 ng/mL) on the expression of E-cadherin (molecular weight, 97 kDa), N-cadherin (molecular weight, 100 kDa), vimentin (molecular weight, 57 kDa), p-Smad2 (molecular weight, 52 kDa) and Smad7 (molecular weight, 51 kDa) by Western Blots. N. Quantitative analysis of treatment of Eca109 cells with TGF-β1 (1, 5, or 10 ng/mL), E-cadherin, N-cadherin, vimentin, P-Smad2 and Smad7 expression levels; Y axis: banding densities of test marker versus β-actin. Data are expressed as a significant change relative to the control. Each bar represents the mean±s.d. *, P<0.05, **, P<0.01. (DOC) [file pone.0112300.s001.doc]

**Supplemental Figure legends**

| **Table S1: Dilution, pretreatment, immunostaining, positive controls, and source for the primary antibodies** | | | | | |
| --- | --- | --- | --- | --- | --- |
| Antibody | Dilution and condition | Pretreatment | Immunostaining | Positive control | Source |
| E-cadherin | 1:100 4°C overnight | PCA CB | Envision | Breast ductal  carcinoma | DAKO, Glostrup, Denmark |
| vimentin | 1:800 4°C overnight | PCA CB | Envision | [fibroma](app:ds:fibroma) | DAKO, Glostrup, Denmark |
| N-cadherin | Ready to use  ,4°C overnight | PCA CB | Envision | Lung cancer | Abcam, Cambridge, United Kingdom |
| TGF-β1 | 1:100 4°C overnight | PCA CB | Envision | Breast cancer | Santa Cruz Biotechnology, California, USA |
| TGF-βRII | 1:100 4°C overnight | PCA CB | Envision | Breast cancer | Santa Cruz Biotechnology, California, USA |
| p-Smad2/3 | 1:100 4°C overnight | PCA CB | Envision | Wounded skin | Santa Cruz Biotechnology, California, USA |
| Note: PCA CB, pressure cooker heating in citrate buffer (0.01M, pH 6.0) | | | | | |


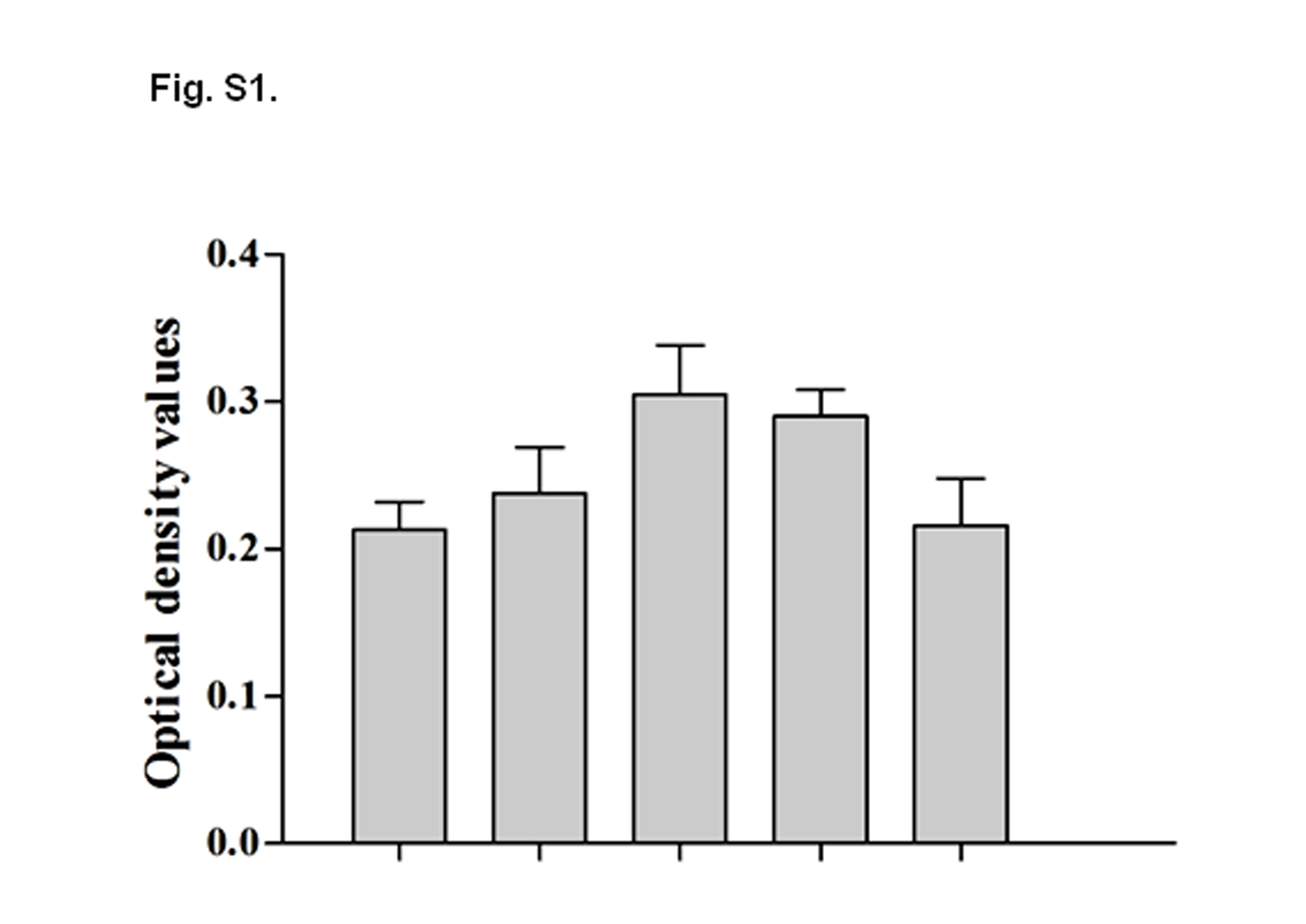


**Fig. S1.** ESCC cells treated with TGF-β1 (0, 1, 5, 10, 15 ng/mL) for 36 hrs. Quantitative analysis of MTT showed no significant differences among different doses.


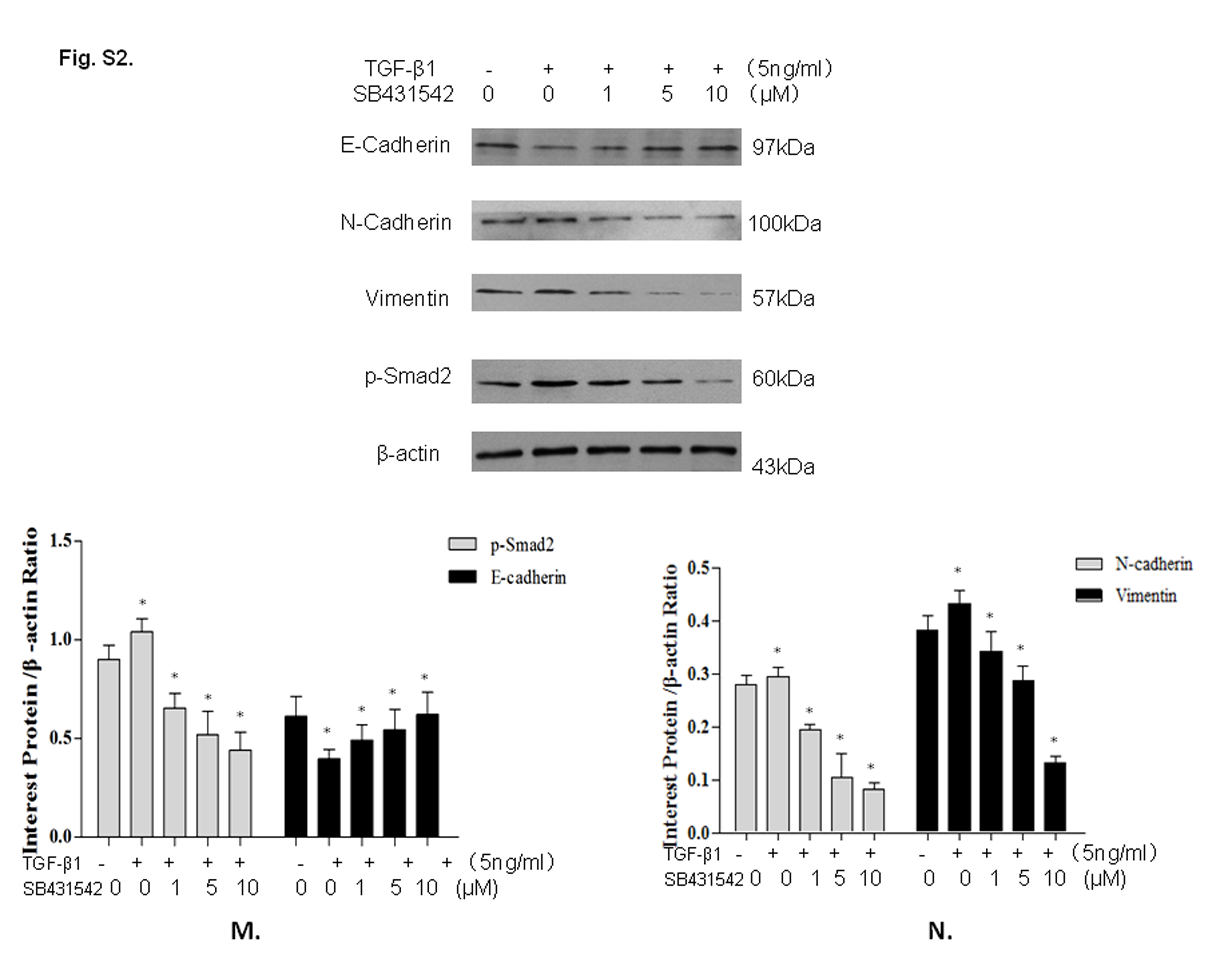


**Fig. S2.** Western blot analysis of E-cadherin, N-cadherin, vimentin, P-Smad2 protein expression in ESCC cells treated with 5ng/ml TGF-β1 in the presence of 0, 1, 5, 10 μM SB431542. **M.** Effects of treatment of Eca109 cells with TGF-β1 (1, 5, or 10 ng/mL) on the expression of E-cadherin (molecular weight, 97 kDa), N-cadherin (molecular weight, 100 kDa), vimentin (molecular weight, 57 kDa), p-Smad2 (molecular weight, 52 kDa) and Smad7 (molecular weight, 51 kDa) by Western Blots. **N.** Quantitative analysis of treatment of Eca109 cells with TGF-β1 (1, 5, or 10 ng/mL), E-cadherin, N-cadherin, vimentin, P-Smad2 and Smad7 expression levels; Y axis: banding densities of test marker versus β-actin. Data are expressed as a significant change relative to the control. Each bar represents the mean±s.d. *, *P*<0.05.
